# Antimicrobial Effect of *Picea abies* Extracts on *E. coli* Growth

**DOI:** 10.3390/molecules24224053

**Published:** 2019-11-09

**Authors:** Nabil Haman, Ksenia Morozova, Giustino Tonon, Matteo Scampicchio, Giovanna Ferrentino

**Affiliations:** Faculty of Science and Technology, Free University of Bozen-Bolzano, Piazza Università 1, 39100 Bolzano, Italy; nabil.haman@unibz.it (N.H.); ksenia.morozova@unibz.it (K.M.); giustino.tonon@unibz.it (G.T.); giovanna.ferrentino@unibz.it (G.F.)

**Keywords:** *Picea abies*, supercritical fluid extraction, isothermal calorimetry, antimicrobials

## Abstract

This study aims to investigate the effect of essential oils extracted from wood residues of *Picea abies* on the growth of *Escherichia coli*. The essential oils were extracted by supercritical carbon dioxide, leading to a yield of 3.4 ± 0.5% (*w*/*w*) in 120 min. The antimicrobial effect was tested at 37 °C by isothermal calorimetry. The heat-flow (dq/dt vs. time) was integrated to give a fractional reaction curve (α vs. time). Such curves were fitted by a modified Gompertz function to give the lag-time (λ) and the maximum growth rate (µmax) parameters. The results showed that λ was linearly correlated with *E. coli* concentration (λ = 1.4 h/log (CFU/mL), R^2^ = 0.997), whereas µmax was invariant. Moreover, the overall heat was nearly constant to all the dilutions of *E. coli*. Instead, when the essential oil was added (with concentrations ranging from 1 to 5 mg/L) to a culture of *E. coli* (10^4^ CFU/mL), the lag-time increased from 14.1 to 33.7 h, and the overall heat decreased from 2120 to 2.37 J. The results obtained by the plate count technique were linear with the lag-time (λ), where (λ = −7.3 × log (CFU/mL) + 38.3, R^2^ = 0.9878). This suggested a lower capacity of *E. coli* to metabolize the substrate in the presence of the essential oils. The results obtained in this study promote the use of essential oils from wood residues and their use as antimicrobial products.

Academic Editors: Vincenzo De Feo and Raphaël E. Duval

## 1. Introduction

Currently, nutraceutical products formulated in order to decrease human diseases are receiving increasing attention. Nutraceuticals are also called functional foods and are defined as food products, that are part of the usual diet having beneficial effects that go beyond a basic nutritional function. Among the several nutraceutical products, those enriched with natural extracts such as ginger, onion, echinacea, liquorice, okra, tea, and turmeric have been receiving great attention for their beneficial effects on health [1,2].

Extracts from coniferous tree species are generating new interest in scientific communities for their potential use in food, medicine, and cosmetics. Among others, the residues of Norway spruce (*Picea abies*) were recently considered an important source of bioactives with potential antimicrobial activity [3,4]. Their inhibitory action includes the degradation of the wall of microbial cells, the damage of the cytoplasmic membrane and membrane proteins, the leakage of cell contents, the coagulation of cytoplasm, and depletion of proton motive force [5]. The main bioactive compounds responsible for their antimicrobial effect are ranked as follows: aldehydes > ketones > alcohols > esters > hydrocarbons. Some studies also identified single compounds responsible for antimicrobial activity such as α-terpineol, δ-3-carene, geranyl acetate, borneol, α and β-pinene, limonene, α-terpinene, γ-terpinene, β-ocimene, bornyl acetate, 1,8- cineole, α-phellandrene, p-cymene, linalool, γ-muurolene, α-humulene, and cadinene [6,7].

However, the bioactivity of such compounds can be greatly influenced by the technology applied for their extraction. Extraction with organic solvents constitutes one of the most commonly used technologies and is largely affected by the polarity of the solvent used, the time of extraction, and the way the contact between the solvent and the matrix is carried out.

Recently, Salem et al. reported that extracts of *Picea abies* obtained by extraction with methanol showed antimicrobial activities over a number of Gram positive and negative microorganisms [8]. However, Vainio-Kaila et al. showed that acetone extracts of *Picea abies* showed a negligible inhibition against the growth of *E. coli*, suggesting that water soluble extracts should be preferred [9]. Conversely, Tanase et al. concluded that the hexane soluble fraction obtained from supercritical carbon dioxide extract of *Picea abies* had antibacterial activity for both Gram positive and negative bacteria. Instead, the ethanol soluble fraction showed no antibacterial potential [10].

Such controversies could be ascribed to the type of antimicrobial test used. Very often, the results of antimicrobial activity are based on antibacterial bioassays such as disk diffusion, well diffusion, and broth or agar diffusion, which are official methods that are well known and commonly used. As pointed out by Balouiri et al. [11], such tests have gained popularity because of their simplicity, low cost, the ability to test an enormous number of microorganisms and antimicrobial agents, and the ease of interpreting the results provided. However, they also suffer from several drawbacks. First, they are unable to distinguish between bactericidal and bacteriostatic effects. Second, the amount of antimicrobial agent that diffused in the agar medium cannot be accounted. Third, the diffusion of the extract into the agar plate may be different depending on the hydrophilic/hydrophobic nature of the extracts.

An alternative technique that has been recently used to analyze microbial growth is isothermal calorimetry. Isothermal calorimetry has proven to be a highly efficient and versatile tool in several fields of microbiology and food science. This technique provides a continuous real-time electronic signal that is proportional to the amount of heat being produced by microorganisms during their metabolic activity [12,13]. The resulting curve obtained by plotting the rate of heat generated by the process vs. time is a thermogram whose shape can be translated into useful microbiological parameters, such as the lag-time or maximum growth rate, through different fitting growth models [14,15]. The use of isothermal calorimetry could extend the current knowledge on the antimicrobial nature of wood residue extracts.

Accordingly, this work aims to investigate the antibacterial effect of extracts from *Picea abies* on the growth of *Escherichia coli*. The antibacterial activity of the extracts will be evaluated by isothermal calorimetry. The heat-flow (dq/dt vs. time) curves will be first integrated to give a fractional reaction curve (α vs. time) and then fitted by a modified Gompertz function. The lag-time (λ) and the maximum growth rate (*µ_max_*) parameters will be obtained and compared to provide evidence of the antibacterial activity of *Picea abies* extracts. Moreover, this work will also demonstrate the use of isothermal calorimetry as an excellent analytical tool to determine the antimicrobial properties of natural extracts.

## 2. Results and Discussion

### 2.1. Thermokinetic Parameters of E. coli Growth by Isothermal Calorimetry

**Figure 1:**
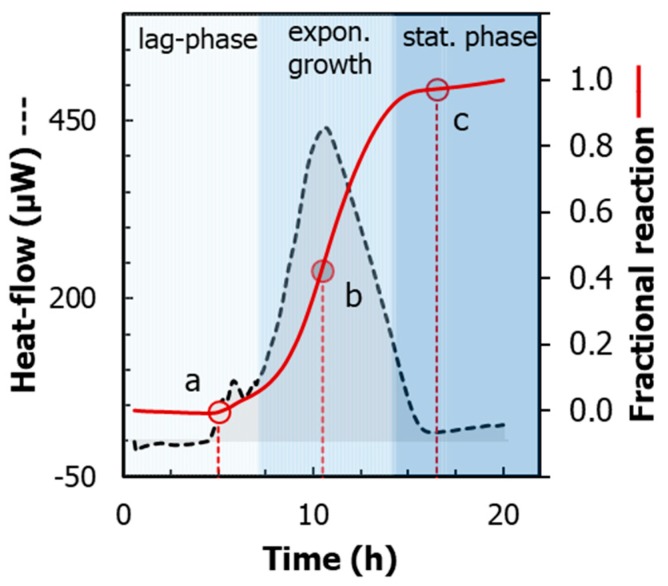
Heat-flow traces of *E. coli*, growing on yeast broth at pH 7.2 and 37 °C in the presence of air in the headspace of the glass ampoule (dashed line). Heat evolved during growth (solid line) was obtained by integrating the area delimited by the heat-flow profile. Red circles indicate, respectively: (**a**) onset time of the exponential growth; (**b**) maximum growth rate; (**c**) total heat. Shown also with different shades of color intensities are the distinct steps of microbial growth, respectively, lag phase, exponential growth, and stationary phase.

Figure 1 (dotted line) shows a calorimetric trace recorded during the aerobic growth of *E. coli* at 37 °C, when all nutrients were available ad libitum. The heat-flow trace (dq/dt vs. time) follows a characteristic pattern that can be qualitatively explained in three distinctive steps:

1. an initial lag-time, where the rate of heat generation is negligible;
2. an exponential rise of the heat-flow signal, indicating the metabolic growth of the cells;
3. a rapid decay of the heat-flow signal, which occurs when either oxygen or substrates limit further growth.

A simple way to characterize these three steps quantitatively is towards the definition of the fractional reaction (α), which is the fraction that changes progressively from reactants (α = 0.00) to products (α = 1.00). From the cumulative integral of the heat-flow curve, α can be easily derived by dividing the resulting cumulative heat (q_t_) by the overall heat (Q_tot_) [14]:

(1) $$\alpha =1-\frac{m_{t}}{m_{0}}=\frac{q\left(t\right)}{Q_{\mathrm{tot}}}$$

An example of a fractional reaction curve is displayed in Figure 1 (solid line). The curve is sigmoidal, and it allows determining two important characteristics of the microbial growth, respectively:

(1) lag-time period (λ), which corresponds to the initial period between the starting of the experiment and the onset-time of the exponential growth (Figure 1, Point a);
(2) maximum growth rate (*μ_max_*), which corresponds to the highest slope observed along the heat-flow curve (Figure 1, Point b).

**Figure 2:**
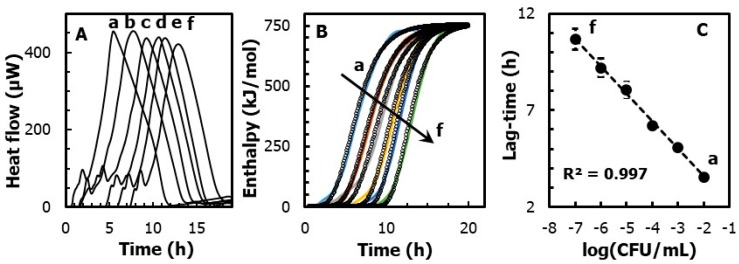
(**A**) Heat-flow traces of *E. coli*, at a concentration from (**A**) 10^7^ to (f) 10^2^ log(CFU/mL). (**B**) Heat vs. time curves obtained by integrating the area delimited by the heat-flow profile. Shown also as dotted points is the fitting of the experimental data obtained by the Gompertz function. (**C**) Linear regression of the lag phase, obtained from the Gompertz fitting function, as a function of the *E. coli* concentration (in CFU/mL).

This approach is exemplified in Figure 2A, where different heat-flow curves are recorded from ampoules containing increasing concentrations of *E. coli* (from 10^2^ to 10^7^ log(CFU/mL)). By using Equation (1), Figure 2B plots the changes of the fractional reaction vs. time. The resulting sigmoidal curves were fitted by an iterative least squares routine. The fitting was performed with the following modified Gompertz function [16]:

(2) $$\alpha =\exp\left[-\exp\left(\mu_{\max}\cdot e\right)\left(\lambda -t\right)+1\right]$$

where λ is the lag phase, *μ_max_* is the maximum growth rate, and *e* is Euler’s number.

It should be noted that, regardless of the concentration of *E. coli*, the maximum growth rate is nearly constant and equal to *µ_max_* = 142 ± 17 mol kJ^−1^ h^−1^. Instead, the lag phase duration is shorter for higher concentrations of microorganisms. In detail, Figure 2C shows that the lag-time is inversely proportional (R^2^ = 0.997) to the concentrations of *E. coli*, following a slope equal to 1.4 h/log(CFU) and an intercept of 0.71 ± 0.1 h. In practice, within the observed range from 10^2^ to 10^7^ CFU/mL of *E. coli*, the lag phase significantly increases (*p* < 0.05) from 3.5 ± 0.5 to 11 ± 0.3 h.

Furthermore, the overall heat (Q_tot_) led to the energy that was generated by the microorganism during the anabolic and catabolic processes. This value can be easily obtained as the area under the heat-flow curve. Overall, Figure 2 shows that, when *E. coli* was grown on tryptone agar substrate, the overall heat generated was nearly constant for all the dilutions considered. The statistical analysis performed on the values revealed no significant differences (*p* < 0.05) between the heat-flows obtained during the growth of *E. coli* at a concentration from 10^7^ to (f) 10^2^ log(CFU/mL). This suggests that the growth of *E. coli* in our system was driven only by the initial number of cells, and it was not complicated by other co-current chemical or physical process (i.e., cell aggregation, cell sedimentation, substrate denaturation, etc.).

### 2.2. Extraction by Supercritical Carbon Dioxide

In this section, we investigate the capacity of essential oils of *Picea abies* to inhibit the microbial growth of *E. coli*. The oils were extracted from *Picea abies* wood residues by a supercritical CO_2_ pilot plant. The extraction included the use of 10% ethanol as the co-solvent. The extraction led to a yield of 3.4 ± 0.5% (*w*/*w*) in 120 min. This result was superior to the yield generally achievable by conventional methods, including solvent extraction and steam distillation [17]. Conventional methods suffer from several shortcomings, such as a large amount of organic solvents needed, losses of some volatile compounds, low extraction efficiency, degradation of unsaturated compounds, and toxic solvent residue in the extract [18,19,20]. Instead, the mild conditions used with supercritical carbon dioxide extraction (45 °C at 20 MPa,) limited the aforementioned drawbacks. The chemical composition of the extract from *Picea abies* was determined by HPLC-HRMS analysis and reported in Table 1.

### 2.3. Microbial Growth Inhibition and Metabolic Activity Inhibition

**Figure 3:**
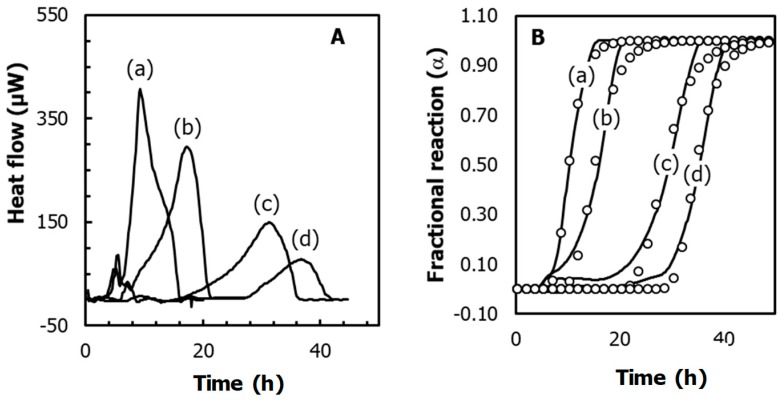
Effect of different concentrations of *Picea abies* extracts: (a) 0 mg/L, (b) 1 mg/mL, (c) 3 mg/mL, and (d) 5 mg/mL, on (**A**) the heat-flow calorimetric traces and (**B**) the fractional reaction. Experimental conditions as in Figure 2.

Figure 3A shows the calorimetric raw data obtained during the growth of *E. coli* in the presence of increasing concentrations of essential oils. The most evident qualitative changes were:

(1) the heat-flow peak was shifted to longer times;
(2) the exponential rise of the signal became less pronounced;
(3) the calorimetric peak area was reduced.

This evidence can be turned into quantitative information through the analysis of the heat curve, as previously described. In detail, Figure 3B shows the fractional reaction curve and the corresponding fitting of the data based on the Gompertz function.

The fitting parameters are reported in Table 2. The results confirmed that the concentration of essential oil was linearly correlated with the lag-time (R^2^ = 0.99). However, the concentration had a negligible effect on the maximum growth rate, which had an average value of 0.14 ± 0.03 h^−1^ (*p* > 0.05). As demonstrated previously, longer lag-times correspond to a lower concentration of *E. coli*. Thus, the effect of essential oil to extend the lag-time may reflect its capacity to inhibit the bacterial growth. A strong correlation (R^2^ = 0.98) was observed between the lag-time (λ) obtained from the isothermal calorimetry experiments and the logarithm of the colonies of *E. coli* determined by traditional plate count at each extract concentration. The inhibition capacity of *Picea abies* extracts against pathogenic and spoilage microorganisms was reported previously [21,22]. Compounds such as vanillic acid and taxifolin, which were contained in the extracts [10], have demonstrated some capacity to accelerate the death of *E. coli* [23]. However, the mode of action of such components is still unclear. Other studies on essential oils from oregano and thyme demonstrated that carvacrol, the most representative active in the oil [24], exerted some antimicrobial action by the disintegration of the outer membrane of Gram negative bacteria, releasing lipopolysaccharides and increasing the permeability of the cytoplasmic membrane to ATP [25]. It is likely that the essential oils from *Picea abies* could explain their bactericidal activity with a similar mechanism. However, further studies are needed to confirm the microbial growth inhibition of essential oils from *Picea abies*.

### 2.4. Metabolic Activity Inhibition

The third effect of essential oil on the microbial growth of *E. coli* was to reduce the calorimetric area. In general, the overall heat (Q_tot_) of a metabolic process is proportional to the amount of substrate (*n*) that has been consumed:

(3) $$Q_{\mathrm{tot}}={\Delta H}_{\mathrm{met}}\cdot n$$

Consequently, the decrease of the overall heat observed when the essential oil is added reflects a lower capacity of the cell to metabolize the substrate and provide specific information on the capacity of the essential oil to inhibit the cell metabolism. Such activity is expressed as:

(4) $$\%\;\mathrm{Metabolic}\;\mathrm{inhibition}=100\cdot \left(1-\frac{Q_{i}}{Q_{0}}\right)$$

where Q_0_ is the overall heat measured in absence of essential oils and *Q_i_* is the overall heat measured in the presence of the essential oil. The results are also reported in Table 2. What is striking is that the minimum amount of extract used (1 mg/mL) was able to reduce the metabolic activity by about 90%. This result was excellent if compared with the antimicrobial activity of other essential oils against Gram negative bacteria. For instance, Gram negative *E. coli* and *Salmonella typhimurium* were tolerant to several essential oils extracted from carrot seed, grapefruit, lemon, onion, and parsley [26]. Such resistance was attributed to the complexity of their double layer cell membrane, compared with the single layer membrane of Gram positive bacteria. Overall, the essential oils extracted from *Picea abies* by supercritical carbon dioxide showed antimicrobial activity, regardless of the protective barrier exerted by their membrane.

## 3. Materials and Methods

### 3.1. Preparation of Wood Extracts

Norway spruce (*Picea abies*) residues were collected locally in the region of South Tyrol (Italy). Before using, they were ground to reach particle sizes in the range of 300–800 μm. The fine powder had a final moisture content of 7.8% on a dry basis and a water activity of 0.4.

### 3.2. Extraction of Essential Oil by Supercritical Fluid 

**Figure 4:**
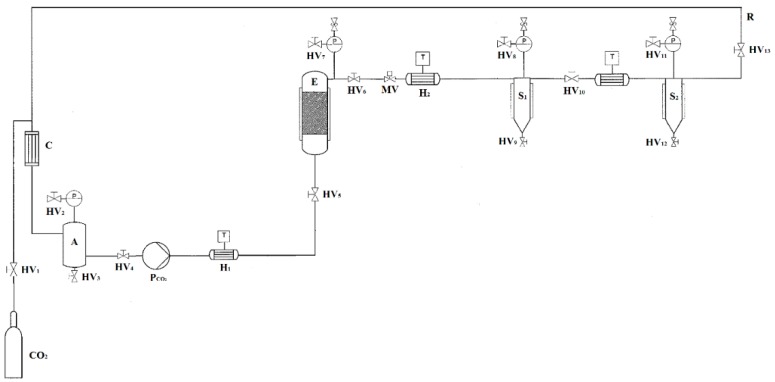
Flowsheet of the supercritical carbon dioxide pilot scale apparatus. CO_2_: storage tank; E: extraction vessel; S#: separators; H#: heat exchangers; C: condenser; HV#: hand valves; MV: membrane valve; P_CO2_: diaphragm pump; T: thermocouples; P#: manometers; R: CO_2_ recirculation; A: liquid storage tank.

Extraction from the residues of *Picea abies* was carried out using a supercritical fluid extractor at pilot scale, as shown in Figure 4 (Superfluidi s.r.l., Padova, Italy). The high pressure vessel (1 L volume) contained an extraction basket of 800 mL, closed with porous stainless steel mesh filters. The CO_2_ was pressurized by a high pressure diaphragm pump (Lewa LDC–M–9XXV1, Milano, Italy) with jacketed heads for cooling. The flow rate of CO_2_ was set to 2 L/h. The system was equipped with a high pressure tank for CO_2_ storage to be recirculated and further reused. About 80 ± 1 g of wood powder, with the addition of 10% (*w*/*w*) of ethanol, was filled inside the extractor. Preliminary experiments were performed to identify the process conditions to achieve the highest extraction yield. A fixed ethanol percentage equal to 10% (*w*/*w*) was considered to increase the concentration of antioxidants in the extracts. A low CO_2_ flow rate of 2 L/h was selected in order to ensure a long residence time of the solvent and a prolonged contact between the sample and the solvent. The effect of the pressure from 10 to 30 MPa, temperature from 35 to 50 °C, and time from 10 to 180 min was tested to find the optimal conditions in terms of the highest yield, expressed as the ratio of the amount of extract to the amount of wood residues placed in the high pressure vessel. Extractions were performed in duplicate. The highest yield was 3.4 ± 0.5% (*w*/*w*), obtained for the conditions of 45 °C, 20 MPa and 120 min of extraction time.

### 3.3. Antimicrobial Activity of Extracts

#### 3.3.1. Test Microorganisms and Growth Media

To assess the antimicrobial activity of spruce extracts from *Picea abies*, *Escherichia coli* (ATCC 25922) was chosen as the test microorganism. The strain was obtained from Agenzia Provinciale per l’Ambiente e la Tutela del Clima (Bolzano, Italy) and stored in tryptone soy broth (TSB)/glycerol (20:80 *w*/*w*) at −80 °C until needed. For experimental use, the stock cultures were maintained on tryptone soy agar (TSA) slants at 4 °C and refreshed monthly.

#### 3.3.2. Antimicrobial Activity by Isothermal Calorimetry

The antimicrobial activity of spruce extracts was tested by isothermal calorimetry. Prior to each experiment, a loopful was transferred to 10 mL of TSB and incubated at 37 °C for 18 h for *E. coli* to obtain fresh early-stationary phase cells. These bacteria suspensions (approximately 10^8^ CFU/mL) were serially diluted in TSB to reach a final concentration of 10^4^ CFU/mL to which the spruce extracts were added. Spruce extracts were also diluted in sterile TSB and then transferred to the bacterial cultures, obtaining a final concentration of extracts equal to 1, 3, and 5 mg/mL. For each concentration of spruce extract, samples were prepared in triplicate. Each sample was transferred to 4-mL sterile stainless-steel vials, closed hermetically with silicone septa, lowered into the thermal equilibration position, and left for 15 min. The heat-flow rates produced during the microbial growths were recorded at an interval of 10 s in isothermal conditions at 37 °C. Data were acquired in triplicate and the calorimetric traces reported in terms of mean values.

#### 3.3.3. Antimicrobial Activity by the Plate Count Technique

The antimicrobial activity of the extract was also checked by the plate count technique following the method used by Tanase et al. [27]. Prior to each experiment, a loopful of *E. coli* was transferred to 10 mL of TSB and incubated at 37 °C for 18 h to obtain fresh early-stationary phase cells. These bacteria suspensions (approximately 10^8^ CFU/mL) were serially diluted in TSB to reach a final concentration of 10^4^ CFU/mL. Spruce extracts were diluted in sterile TSB agar obtaining a final concentration equal to 1, 3, and 5 mg/mL. Then, 100 μL aliquots of the bacteria final concentration were spread on the surface of the TSB agar plate. The aliquot was streaked over the entire sterile agar surface, rotating the plate to ensure a homogeneous distribution of the inoculum. The plates were allowed 3 to 5 min to dry the excess moisture. The plates were labeled and placed in an incubator set to 37 °C. After 24 h of incubation, each plate was examined, and the colony grown on the solid agar was determined. A total of three plates were used for each extract concentration and the results expressed as the average and standard deviations.

### 3.4. Characterization of Picea abies Extract by HPLC-HRMS

The characterization of the phenolic compounds of *Picea abies* extract obtained by supercritical fluid extraction was performed by HPLC-HRMS. The system used consisted of a Thermo Sci. Q-Exactive Orbitrap HRMS instrument coupled to an Ultimate 300 UHPLC instrument. The separation of the antioxidant compounds was done at a flow rate of 0.2 mL min^−1^ with an Accucore RP-MS LC column (100 mm × 2.1 mm i.d., 2.6 μm) with a corresponding pre-column (Thermo Scientific). The mobile phase consisted of a combination of Solvent A (water with the addition of 20 mM ammonium formate, 0.1% formic acid *v*/*v*) and B (acetonitrile with 0.1% formic acid). The gradient was set as follows: from 5% B at 0 min to 25% B (*v*/*v*) at 21 min, then to 95% B at 22 min until 27 min, to 5% at 28 min, followed by a re-equilibration step (5% B) from 28 to 32 min. For Full-MS analysis, the mass spectrometer was operated in negative ionization mode using the following conditions: sheath gas at 20 (arbitrary units), aux gas at 5 (arbitrary units), aux temperature 250 °C, spray voltage at ±3.5 kV, capillary temperature at 320 °C, and S-lens radio frequency (RF) at 65 °C. The mass range selected was from 100 to 1000 *m*/*z* with a full-MS set resolution of 70,000 at *m*/*z* 200, automatic gain control (AGC) target at 1 × 10^6^, and max. injection time of 175 ms. The MS^2^ measurements of the selected ions were performed with a resolution of 17,500 and AGC target set at 5·10^5^. Individual phenolics were identified on the basis of their retention time, UV absorbance at 280 nm, and MS^2^ spectra comparison with external analytical standards and the mzCloud Advanced Mass Spectral Database. The correlation of chemical compounds’ relative abundances and integration of the area under each peak were done using Compound Discoverer 2.1 software (Thermo Scientific, Milano, Italy).

### 3.5. Statistical Analysis

All the results are expressed as means ± standard deviation (SD) of three parallel measurements. The results were statistically evaluated by an analysis of variance (ANOVA), using XLSTAT software Version 2016.02.28014 (Addinsoft, New York, NY, USA) in order to detect significant differences between values of the overall heat (Q_tot_) and the lag-time (λ) obtained with different concentrations of *E. coli* and with the addition of *Picea abies* extract. The significant differences (*p* < 0.05) were analyzed by the Tukey test.

## 4. Conclusions

Overall, the results unambiguously provided the antibacterial activity of *Picea abies* extract on the growth of *E. coli*. The extracts inhibited not only the growth, but also interfered with the metabolic activity of the microorganism. This behavior was relevant if compared with that of other essential oils previously reported in the literature, whose sensitivity towards *E. coli* was very limited. Further studies are needed in order to perform antimicrobial activity test on individual compounds detected in the extract and to identify those responsible for this action. The results obtained by isothermal calorimetry were also compared with those obtained by the plate count technique. A strong correlation (R^2^ = 0.98) was observed between the lag-time (λ) and the logarithm of the colonies of *E. coli*. These results confirmed that isothermal calorimetry can be used to follow continuously in real time the microorganisms during their metabolic activity. The technique can provide results to support those of the official methods (such as disk diffusion, well diffusion, and broth or agar diffusion). It generates a large amount of data, which can be used to model the metabolic activity of microorganisms. However, a preliminary correlation of the results with those of the classical methods is required. Moreover, compared to the official methods, which are simple, low cost, and provide an easy interpretation of the results, the technique has some limitations due to the cost associated with the instrument and the initial comprehension of the microbial parameters obtained by the thermograms.

## Figures and Tables

**Table 1:** Composition of *Picea abies* extract determined by HPLC-HRMS analysis in negative ionization mode.

Compound	Formula	[M − H]^−^ Theoretical	[M − H]^−^ Measured	Δmi * (Da)	Δppm	RT in ESI (−)	Area × 10^6^
Cinnamic acid	C_9_H_8_O_2_	147.0451	147.0452	0.0001	0.7	4.9	2.03 ± 0.1
Protocatechuic acid	C_7_H_6_O_4_	153.0193	153.0192	−0.00001	−0.1	3.44	1.5 ± 0.1
*p*-Coumaric acid	C_9_H_8_O_3_	163.0401	163.0401	−0.00002	−0.1	11.7	1.6 ± 0.1
Gallic acid	C_7_H_6_O_5_	169.0142	169.0143	0.0001	0.6	1.96	4.1 ± 0.3
Ferulic acid	C_10_H_10_O_4_	193.0506	193.0507	0.0001	0.4	4.6	0.60 ± 0.13
(+)-Catechin	C_15_H_14_O_6_	289.0718	289.072	0.0002	0.7	6.7	1.6 ± 0.3
Dihydroquercetin (Taxifolin)	C_15_H_12_O_7_	303.0513	303.0513	0.0000	0.0	14.74	59 ± 2
Hydroxypinoresinol	C_20_H_22_O_7_	373.1293	373.1293	0.0000	0.0	18.4	312 ± 23
Astringin	C_20_H_22_O_2_	405.1191	405.1196	0.0005	1.2	11.16	16 ± 1
Isorhapontin	C_21_H_24_O_9_	419.1348	419.1349	0.0001	0.2	18.4	432 ± 56

* Mass measurement accuracy measured in Dalton and defined as difference between the theoretical mass and the measured mass.

**Table 2:** Kinetic parameters obtained from the thermogram of *E. coli* growth and results obtained by the plate count technique.

	Gompertz Function				Metabolism Inhibition	Plate Count
Extract Concentration	Q_tot_	λ	µ_max_	R^2^	100 (1 − Q_1_/Q_0_)	
mg/mL	J	h	H^−1^	-	%	log(CFU/mL)
-	2120 ± 39	9.5 ± 0.6	0.18 ± 0.06	0.999	-	4.11 ± 1.5
1	218 ± 17	14.1 ± 0.3	0.12 ± 0.09	0.995	89.7 ± 0.5	2.91 ± 0.7
3	5.3 ± 0.5	27.3 ± 1.7	0.10 ± 0.02	0.994	99.8 ± 0.2	1.53 ± 0.5
5	2.4 ± 0.2	33.7 ± 1.1	0.12 ± 0.05	0.999	99.9 ± 0.1	0.70 ± 0.3
